# A Miniaturized and Modular Wearable Functional Near-Infrared Spectroscopy (fNIRS) Sensing Module for High-Density Cerebral Hemodynamic Monitoring

**DOI:** 10.3390/bios16040192

**Published:** 2026-03-26

**Authors:** Mengjie Fang, Xinlong Liu, Bowen Ji, Le Li, Kunpeng Gao

**Affiliations:** 1 School of Information and Intelligent Science, Donghua University, Shanghai 201620, China; 2232280@mail.dhu.edu.cn (M.F.); 2232148@mail.dhu.edu.cn (X.L.); 2 National Key Laboratory of Unmanned Aerial Vehicle Technology, Unmanned System Research Institute, School of Artificial Intelligence, Northwestern Polytechnical University, Xi’an 710072, China; bwji@nwpu.edu.cn; 3 School of Life Science and Technology, Northwestern Polytechnical University, Xi’an 710129, China; lile5@nwpu.edu.cn

**Keywords:** fNIRS, modular design, wearable, neurovascular coupling, brain functional imaging, hemoglobin

## Abstract

This study presents a modular and scalable wearable functional near-infrared spectroscopy (fNIRS) system for high-resolution cerebral hemodynamic signal acquisition. The system is based on compact optoelectronic modules and supports mixed measurements using short-separation and long-separation channels, offering good scalability and spatial adaptability. The integrated quartz light guide structure improves optical coupling efficiency between the probe and scalp. A series of in vivo experiments validated system performance. In a forearm arterial occlusion experiment, the system accurately captured concentration changes in oxygenated and deoxygenated hemoglobin during blood flow blockade and reperfusion, with large effect sizes (Cohen’s d > 0.9). In a prefrontal cortex Valsalva experiment, the biphasic response characteristic of neurovascular coupling was successfully resolved. In a 2-back working memory task, the system identified a task-related frequency component (0.0227 Hz) and right-lateralized prefrontal cortex activation (*p* = 0.023). These results demonstrate that the system exhibits a good signal-to-noise ratio and temporal dynamic response, enabling high-resolution mapping of regional hemodynamic changes. This work provides an effective solution for the development of wearable, modular, and high-precision multi-channel fNIRS systems.

## 1. Introduction

Brain–Computer Interface (BCI) technology enables direct information exchange and functional control between the human brain and external devices—such as computers, intelligent prosthetics, and assistive communication systems—by accurately decoding electrophysiological or metabolic signals generated by neuronal activity. This capability provides new technical approaches for restoring communication and motor function in patients with severe motor impairments [1]. Currently, BCI systems have been widely applied in areas such as clinical communication control (e.g., thought-based typing for locked-in syndrome), collaborative interaction with intelligent robots, biomedical engineering (e.g., diagnosis of brain functional disorders), and neurorehabilitation (e.g., post-stroke motor recovery) [2,3].

Among non-invasive BCI modalities, functional near-infrared spectroscopy (fNIRS) has emerged as a promising brain imaging technique due to its distinctive advantages [4,5]. Based on the principle of the hemodynamic response, fNIRS quantifies changes in oxygenated hemoglobin (HbO_2_) and deoxygenated hemoglobin (HbR) concentrations by measuring the absorption and scattering of near-infrared light in cortical tissue. These variations indirectly reflect the spatial and temporal patterns of neural activity. Compared with other neuroimaging techniques, fNIRS provides a balanced trade-off between spatial and temporal resolution: its spatial resolution can reach the centimeter level, allowing precise localization of activation in brain regions such as the prefrontal and motor cortices, while its temporal resolution (hundreds of milliseconds) allows real-time tracking of dynamic neural processes. This balance effectively compensates for the low temporal resolution of functional magnetic resonance imaging (fMRI) and the limited spatial localization of electroencephalography (EEG) [6]. Moreover, fNIRS is non-invasive and does not require scalp penetration or exogenous contrast agents for signal acquisition. The compact and wearable design of fNIRS devices further enables long-term monitoring of brain activity in natural environments, such as during walking or task performance [7].

Owing to these advantages, fNIRS has demonstrated significant value across multiple interdisciplinary fields, including psychiatric assessment (e.g., prefrontal function analysis in depression), language cognition research (e.g., hemispheric differences in native and second language processing), educational neuroscience (e.g., monitoring brain dynamics during learning), and non-invasive BCI development [8,9,10].

Early fNIRS systems primarily relied on mobile cart-based platforms (e.g., Hitachi ETG-4000 series, Shimadzu OM-100 A), where light source and detector modules were connected to scalp-mounted probe arrays via several-meter-long optical fibers [11]. Although such fiber-based systems improved portability compared to large-scale fMRI or costly magnetoencephalography (MEG) setups, they still suffered from inherent limitations. Long optical fibers are prone to bending loss and breakage, leading to signal attenuation and reduced stability under environmental interference. In addition, fiber tethering restricts subjects to remain stationary within a confined space, preventing fNIRS measurements during natural behaviors such as walking or arm movement. These factors limit the applicability of fNIRS in naturalistic and rehabilitation contexts [12,13].

As fNIRS applications expand toward portable monitoring, high spatiotemporal resolution imaging, and multi-region synchronization [14], the limitations of traditional systems have become increasingly evident. These constraints mainly include: (1) limited environmental adaptability—the bulky cart-based structure and fiber tethering hinder the use of such systems in dynamic scenarios such as neurorehabilitation training or outdoor brain monitoring. For instance, during post-stroke rehabilitation, patients are required to perform limb extensions or gait exercises, but conventional systems cannot synchronize brain monitoring with body movements; and (2) insufficient probe flexibility—traditional systems often employ an integrated probe layout with fixed geometry, making it difficult to adjust array density and coverage according to head size or target brain regions. Differences in the prefrontal cortex coverage between children and adults, or between motor and language cortex positioning, further limit flexible high-density imaging of functional brain networks [15,16,17,18].

To overcome these technical limitations, modular design has emerged as a key direction for improving the flexibility, scalability, and environmental adaptability of fNIRS systems. Existing modular solutions integrate core components—such as light source drivers and signal detectors—into compact sensing modules that communicate wirelessly or through flexible cables [15,18]. However, these designs still focus on the independent operation of individual modules. Their channel count is constrained by the optode layout within each module, making it difficult to effectively multiply channel numbers through module combinations for high-density imaging [18]. To address this limitation, we propose a wearable fNIRS system based on inter-module cooperative imaging. Through precise bus communication and timing synchronization, the light sources and detectors of adjacent modules can be freely combined to form measurement channels—ranging from a basic 1 × 2 layout (2 modules) to a full-head 7 × 4 layout (28 modules), with channel counts expandable from 16 to over 64.

This design offers multiple advantages. First, it is highly reconfigurable: users can flexibly adjust the number and arrangement of modules according to research needs. This allows both high-density coverage of a single brain region (e.g., over 64 channels for fine-grained monitoring of the prefrontal cortex) and simultaneous acquisition from multiple regions such as the prefrontal cortex, motor cortex, and temporal lobe through multi-module combinations. Second, it enables cost control: standardized modules can be mass-produced, simplifying the manufacturing process. Damaged modules can be replaced individually, reducing maintenance costs. Third, it enhances portability: the lightweight modules can be fixed using a flexible headband, eliminating the constraints of optical fibers and carts, and making the system suitable for monitoring in dynamic scenarios [19,20].

Based on this concept, we developed a lightweight, modular, and reconfigurable high-density cerebral oximetry device. The system is built around miniaturized sensing modules. Each module integrates two near-infrared light sources (wavelengths: 735 nm and 850 nm, covering the characteristic absorption peaks of hemoglobin) and two photodetectors. The module dimensions are only 20 mm × 20 mm × 8 mm, with a weight of approximately 8 g. Users can customize probe arrays with varying densities—such as 2 × 2 or 4 × 6 layouts—according to experimental requirements. Channel counts can be expanded from 16 to over 64, meeting the monitoring needs of different brain regions and spatial resolutions. Through modular topology optimization, the system achieves a spatial resolution of 8.5 mm (center-to-center distance between adjacent detection points) and a temporal resolution of up to 2 ms. In a working memory task, a measured channel signal-to-noise ratio (SNR) of 6.08 was obtained.

To validate the reliability and practicality of the device, a series of physiological and cognitive experiments was conducted. In a forearm arterial occlusion experiment, blood flow was blocked to induce local hemodynamic responses. The system successfully captured the dynamic changes in HbO_2_ concentration, which decreased within 5 s after occlusion and recovered within 10 s after reperfusion. In a breath-holding experiment, the system successfully captured the typical biphasic response: HbO_2_ concentration briefly decreased at the onset of breath-holding, then continuously increased until the end of the breath-hold, and gradually returned to baseline during recovery. Quantitative analysis of breath-holding events showed a mean decrease in amplitude of 13.86 ± 3.85 μM and a mean increase in amplitude of 38.69 ± 16.21 μM. In a 2-back working memory task, the system effectively differentiated activation patterns in the prefrontal cortex. The ΔHbO_2_ amplitude in the right prefrontal cortex (PFC) (0.316 ± 0.060 μM) was significantly higher than that in the left PFC (0.219 ± 0.041 μM) (paired *t*-test: t(5) = 3.23, *p* = 0.023), validating the right-lateralized activation under high working memory load. These results demonstrate that the system can accurately capture cognitive load-related brain functional changes. Overall, the proposed modular device exhibits reliable dynamic cerebral oxygenation signal acquisition capabilities and can provide high-quality physiological signals for applications such as BCI signal decoding, diagnosis of brain functional disorders, and assessment of neurorehabilitation.

## 2. Materials and Methods

### 2.1. Architecture and Modular Design

**Figure 1:**
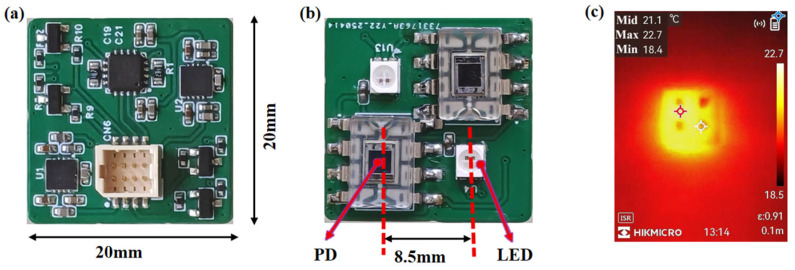
Visualization of the developed fNIRS instrument: (**a**) Top view (functional layer): Layout of signal conditioning circuits and communication modules. (**b**) Bottom view (contact layer): Integration of PD and dual-wavelength LEDs facing the skin surface. (**c**) Thermal image of a single module under operating conditions (maximum temperature: 22.7 °C < 42 °C).

The primary challenge in designing a wearable fNIRS system lies in balancing the demands of miniaturization with the need for layout flexibility. Due to the inability of large, rigid printed circuit board (PCB) to conform to the curvature of the skull, optical coupling failures and discomfort during wear can occur [21]. In this design, a 20 mm × 20 mm miniaturized PCB substrate was employed (Figure 1), ensuring complete integration of electronic components while enhancing the probe’s adaptability to complex cranial contours.

Each functional module adopts a dual-mode optical architecture. The emission unit integrates a constant-voltage-driven dual-wavelength LED light source (735 nm/850 nm, emission area: 2.4 mm × 2.4 mm). The detection unit incorporates a high-sensitivity photodiode (effective sensing area: 2.29 mm × 2.29 mm). This modular design endows the system with dual measurement capabilities. When superficial tissue detection is required, the LED–photodiode pair within the same module enables short-separation measurements (source–detector spacing < 10 mm). When deeper brain tissue signals need to be acquired, adjacent modules can be interconnected to form long-separation measurement channels (source–detector spacing > 20 mm). This configuration establishes a detection framework covering different cortical depths [22,23]. Short-separation channels are intended to probe hemodynamic changes in superficial tissues such as scalp and skull. These signals can serve as physiological nuisance regressors in subsequent analyses to enhance the specificity of cortical signals. The hardware architecture of the proposed system fully supports this functionality.

To accommodate diverse experimental scenarios, the system offers an expandable channel architecture. The basic layer consists of 2 modules (1 × 2 layout) forming the minimal detection unit, which can be incrementally expanded up to 28 modules (7 × 4 layout) through modular topology, creating a sensor network that covers the entire cortical area. Each module operates independently with its own power supply and uses an I^2^C bus for data synchronization. The sampling rate is dynamically configurable from 7.14 Hz to 200 Hz, with a channel density of up to 1.875/cm^2^. This hardware reconfiguration mechanism allows for on-demand configuration and dynamic expansion of measurement channels, meeting the precise monitoring needs of specific brain regions for basic research, while also supporting whole-brain functional imaging with high spatiotemporal resolution.

The modular design enables convenient physical reconfigurability of the system. Users can adjust the probe array layout within minutes by loosening the fixing elastic cap, removing the module, repositioning it onto the target grid node, and finally adjusting the optode–scalp contact. For example, reconfiguring the array from a 1 × 4 prefrontal configuration (used for working memory studies) to a 2 × 2 motor cortex configuration (used for motor function studies) takes approximately 3–5 min. Spatial resolution is determined by the source–detector spacing and arrangement density. With a minimum source–detector spacing of 8.5 mm, the theoretical spatial resolution limit is approximately 4–5 mm (half the spacing). The achievable resolution in practice is influenced by factors such as tissue optical properties and signal-to-noise ratio.

### 2.2. Sensor Block Design

**Figure 2:**
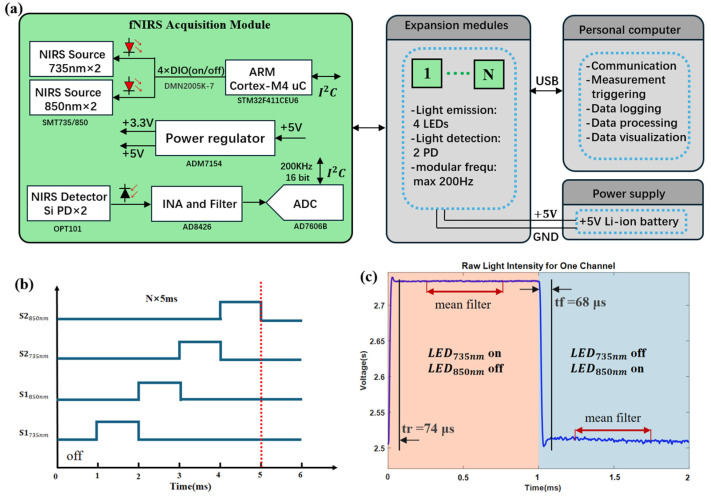
(**a**) Schematic diagram of a single module: each module includes two light sources and two detectors, along with power supply modules, amplification circuits, etc.; (**b**) time-division multiplexing cycle diagram: a single module multiplexes four LEDs for 4 ms and dark measurement for 1 ms. For N modules, the cycle is N × 5 ms; (**c**) photodetector (PD) signal response under LED TDM operation: The orange line represents when the LED at 735 nm is on, and the LED at 850 nm is off, with a rise time of 74 μs; the blue section represents when the LED at 735 nm is off and the LED at 850 nm is on, with a fall time of 68 μs. The red line section represents downsampling with averaging to improve signal quality.

The fNIRS instrument proposed in this paper adopts a modular and reconfigurable design concept (Figure 2a). The system is composed of multiple small square PCB modules. Each functional module integrates a dual-wavelength LED, PD and signal processing circuits. A dual-layer PCB layout is used to achieve physical isolation of functional components: the optical elements are arranged on the bottom layer facing the scalp, while the driving circuits and communication modules are integrated on the top layer. This layered design could reduce electromagnetic interference.

Regarding light source selection, although laser diodes (LDs) offer advantages such as high penetration depth and narrow spectral bandwidth, their high cost, relatively large size, safety concerns, and heat generation limit their suitability for wearable devices. Therefore, this study adopted surface-mount dual-wavelength LEDs (SMT735/850, USHIO OPTO SEMICONDUCTORS, INC, Tokyo, Japan) as an alternative. The selected LEDs emit at 735 nm and 850 nm, positioned on either side of the hemoglobin isosbestic point (805 nm), with an average spectral bandwidth of 10 nm. This wavelength combination enables sensitive detection of oxygenation signals in brain tissue [23,24]. Under constant-voltage driving, the optical output power of the LED is 20 mW at 735 nm and 24 mW at 850 nm.

To meet biosafety requirements, the system strictly adheres to the temperature control standard for skin-contact components (≤42 °C) to prevent thermal damage [25]. To evaluate the actual power consumption and heat generation of the system, we measured the power consumption under different configurations. Under typical operating conditions (sampling rate: 10 Hz), the average power consumption of a single functional module is 295 mW. For a typical 4-module array (commonly used for prefrontal cortex monitoring), the total power consumption is 520 mW. To verify the thermal safety of the system during prolonged wear, we measured the surface temperature of the device using an infrared thermal imager (HM-TPH21Pro-3AQF, Hangzhou, China). Figure 1c shows the thermal imaging result of one module after 30 min of continuous operation. The maximum temperature of all light source–detector modules was 22.7 °C (ambient temperature: 18.4 °C), which is well below the 42 °C safety standard. This preliminarily validates the thermal safety of the system in typical application scenarios.

The light sources employ a time-division multiplexing (TDM) architecture (Figure 2b), with precise temporal control implemented via MOSFET switching circuits driven by an STM32F411CEU6 microcontroller (STMicroelectronics, Plan-les-Ouates, Switzerland). Each light source is driven sequentially with a minimum pulse width of 1 ms, with timing jitter strictly controlled within ±75 μs (Figure 2c) to ensure the stability of light intensity output. The emission cycle for a single module is 4 ms for light emission and 1 ms for dark measurement, with a total cycle time of 5 ms. When expanded to an array of N modules, the system cycle is synchronized and extended to N × 5 ms, ensuring multi-channel data synchronization while effectively controlling overall power consumption.

Detector performance directly affects signal acquisition quality, particularly in miniaturized devices where sensitivity and power consumption must be balanced. In this study, a photodiode (PD) was selected as the core detection component. Photodiodes offer stable operation without requiring precision-regulated power supplies or cooling systems, significantly reducing device size and manufacturing cost. Specifically, the OPT101 photodiode (Texas Instruments, Dallas, TX, USA) was adopted as the core detection element. Its photoelectric response exceeds 0.7 A/W at both 735 nm and 850 nm, enabling efficient capture of weak near-infrared light signals. The built-in transimpedance amplifier architecture integrates photoelectric conversion with signal conditioning. The device also features high common-mode rejection ratio and low dark current, providing excellent anti-interference capability and long-term monitoring stability.

For the microvolt-level raw signals output by the OPT101, which are difficult to acquire directly, the signal was first amplified with a fixed gain of 30 using a dual-channel AD8426BCPZ-R7 (Analog Devices Inc., Norwood, MA, USA) instrumentation amplifier. Its 120 dB common-mode rejection ratio at 10 Hz effectively suppresses environmental noise such as power-line interference. The amplified signal was then passed through a first-order low-pass filter composed of a 10 kΩ resistor and a 100 nF capacitor. The cutoff frequency was designed at 160 Hz to precisely filter out high-frequency electronic noise. Finally, the signal is digitized by an AD7606B (Analog Devices Inc., Norwood, MA, USA) analog-to-digital converter with a maximum sampling rate of 200 kSPS. This configuration allows the collection of 1000 sample points within the 5 ms measurement cycle, and the wavelength signals were averaged and downsampled (Figure 2c) to improve signal quality. Consequently, the system offers a configurable sampling rate range from 7.14 Hz (full 28-module configuration) to 200 Hz (single-module mode), allowing users to balance channel density and temporal resolution according to their research requirements.

### 2.3. Photodetector Probe Structure Design

**Figure 3:**
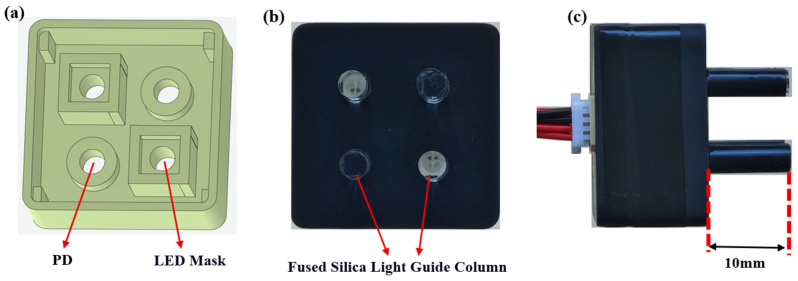
Mechanical packaging of the module: (**a**) Internal view of the module bottom shell: surface spray treatment, with internal light shielding isolation. (**b**) External bottom view of the module shell: the white part represents the quartz light guide rod with a diameter of 3 mm. (**c**) Side view of the module shell: the light guide rod protrudes 10 mm from the shell, improving contact with the brain’s scalp.

The mechanical packaging of the photodetector probe needs to balance both optical performance and wearability comfort. This study achieves the synergistic optimization of these two core requirements through structural design. A modular shell was constructed using 3D printing technology, with the main material being black ABS resin. An integrated 1.2 mm thick light-blocking light guide shield forms the primary optical isolation layer (Figure 3a) [26]. To further enhance light sealing performance, a black zinc-nickel alloy coating was applied to the surface of the shell, creating a dual protection mechanism to ensure that the LED light source and detector are completely enclosed, effectively eliminating interference from ambient light and internal light leakage.

For light transmission, custom-designed quartz glass light guides were connected to both the light source emitters and the detector receivers (Figure 3b). These light guides have a diameter of 3 mm and are made of quartz glass, offering a spectral transmittance of up to 93.4% in the 700–900 nm near-infrared range. The light guide protrudes 10 mm from the housing (Figure 3c), providing two advantages. First, the non-contact optical coupling prevents direct contact between the LED and the skin, avoiding heat accumulation and simplifying disinfection procedures. Second, the extended light guide structure enables stable contact with the scalp, enhancing light transmission performance.

## 3. Results

To systematically evaluate the dynamic response performance and neurovascular coupling detection capability of the proposed fNIRS monitoring architecture in complex physiological environments, a series of standardized in vivo experiments was designed. All experiments were conducted in a standardized laboratory environment shielded from external interference, strictly adhering to ethical guidelines. Prior to the experiments, all participants received an explanation of the experimental procedure and signed a written informed consent form.

### 3.1. Data Processing

The core detection principle of functional near-infrared spectroscopy (fNIRS) technology is based on the neurovascular coupling effect. When the brain is stimulated by external tasks and triggers active neural activity, the concentrations of oxygenated hemoglobin (HbO_2_) and deoxygenated hemoglobin (HbR) undergo dynamic changes. This technology implements optical detection using a light source-detector array fixed on the scalp. The light source emits near-infrared light at specific wavelengths (735 nm/850 nm), which penetrates the skull and brain tissue. The detector receives the emitted light that has been absorbed and scattered by biological tissues, and the hemodynamic response is inverted from the light intensity attenuation.

**Figure 4:**
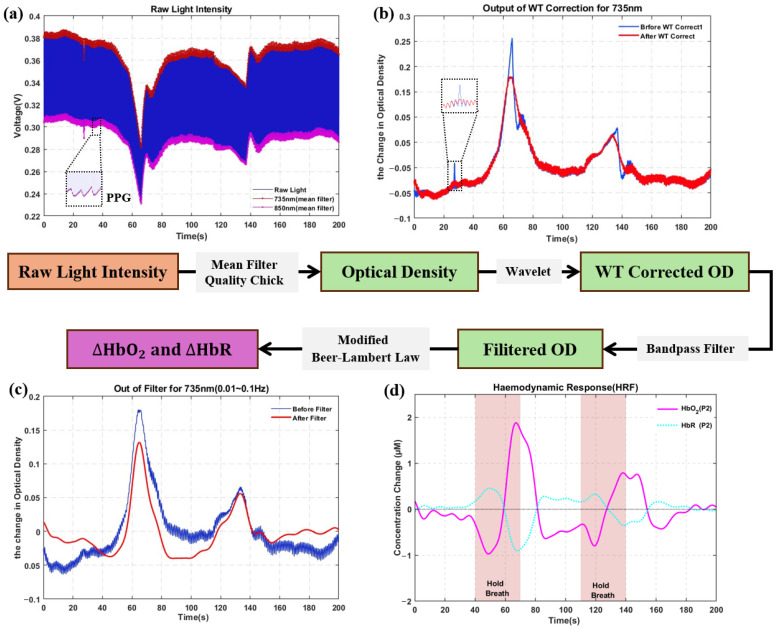
Data processing flow of the Valsalva maneuver experiment for Subject 2: (**a**) Raw optical intensity: The raw optical intensity recorded by one channel. This intensity represents the light detected by the photodetector after passing through biological tissue (red for 735 nm intensity, magenta for 850 nm intensity). The inset shows the photoplethysmographic (PPG) waveform used for heart rate calculation. (**b**) Motion artifact identification and correction (wavelet method): The panel compares the optical density (OD) signal before (blue) and after (red) wavelet-based motion correction. The inset shows the motion-corrected OD signal. (**c**) Filtering: A bandpass filter (0.01–0.1 Hz) is applied to eliminate physiological and high-frequency noise. Compared to the unfiltered signal (blue), the filtered OD signal (red) exhibits improved smoothness and clarity. (**d**) Final hemodynamic response: The processed OD signals are converted into HbO_2_ (red) and HbR (blue) concentration changes via the modified Beer–Lambert law.

Figure 4 illustrates the complete data processing pipeline using the Valsalva breath-holding experiment of Subject 2 as an example. All computations were performed in the MATLAB 2022b environment.

Raw light intensity processing: Raw light intensity signals were acquired using a time-division multiplexing scheme. The time-series data from each detector channel were first segmented (Figure 4a). Mean filtering was applied by extracting the central 0.5 ms interval within each 1 ms sampling window. The red and orange curves correspond to the 735 nm and 850 nm wavelengths, respectively. This approach effectively suppresses high-frequency noise while preserving physiological signal characteristics. The magnified view clearly shows photoplethysmography (PPG) waveforms. PPG is highly sensitive to optical coupling quality—poor coupling causes the waveform to be submerged in noise. The presence of the PPG waveform objectively confirms that the light guide design achieves efficient and stable optical coupling [27,28]. In addition, a 20 s segment free of tasks and motion artifacts was extracted from resting-state data. The standard deviation of HbO_2_ concentration fluctuations was calculated as baseline noise, yielding a value of 0.3419 μM, indicating good signal stability of the system.

Motion artifact identification and correction: Raw data were converted to optical density (OD) parameters using the HOMER3(v1.80.2) toolbox [29,30]. Motion artifacts were identified using a composite threshold detection algorithm based on moving standard deviation and amplitude changes implemented in HOMER3. Specifically, for the OD time series of each channel, a time window tMotion = 0.5 s was used. If the signal standard deviation exceeded a threshold SDThresh = 5.0, or the peak-to-peak amplitude exceeded a threshold AMPThresh = 0.5, the interval tMask = 1 s before and after that time point was flagged as a motion artifact-contaminated segment [31]. Motion artifact correction employed a wavelet filtering method. The OD signal containing artifacts was first subjected to continuous wavelet transform. Outlier coefficients were identified based on the statistical distribution of wavelet coefficients using the interquartile range threshold method (iqr = 1.5, turnon = 1) and set to zero. The signal was then reconstructed through inverse wavelet transform [32,33]. Figure 4b compares the OD signal before and after correction. Severe fluctuations were effectively suppressed while task-related hemodynamic responses were preserved.

Physiological noise filtering: A zero-phase distortion Butterworth bandpass filter (0.01–0.1 Hz) was applied to remove physiological noise components, including heartbeat (1–1.5 Hz), respiration (0.2–0.5 Hz), and Mayer waves (~0.1 Hz). Figure 4c shows the filtered signal (red curve) compared to the raw data (blue curve). The filtered signal exhibits notably improved signal-to-noise ratio while preserving task-related features.

Hemoglobin concentration calculation: The modified Beer–Lambert law (MBLL) was applied to convert light attenuation into concentration changes for HBO_2_ and HbR (Figure 4d) [34]. For the dual-wavelength measurement system employed in this design (λ1 = 735 nm, λ2 = 850 nm), the following system of equations was established to relate light attenuation to concentration changes:

(1) $$\left\{\begin{array}{c} {\Delta OD}_{\left(\Delta t,\lambda 1\right)}\;=\;\left({Ԑ}_{{Hbo}_{2},\lambda 1}\;\cdot \Delta \left[{HbO}_{2}\right]+{Ԑ}_{HbR,\lambda 1}\cdot \Delta \left[HbR\right]\right)\;\cdot \;{DPF}_{\lambda 1}\;\cdot \;L\\ {\Delta OD}_{\left(\Delta t,\lambda 2\right)}\;=\;\left(\;{Ԑ}_{{Hbo}_{2},\lambda 2}\;\cdot \Delta \left[{HbO}_{2}\right]+{Ԑ}_{HbR,\lambda 2}\cdot \Delta \left[HbR\right]\right)\;\cdot \;{DPF}_{\lambda 2}\;\cdot \;L \end{array}\right.$$

By matrix inversion, the changes in hemoglobin concentrations were computed as follows:

(2) ΔHbRΔHbO2=(L)−1ԐHbR,λ1ԐHbo2,λ1ԐHbR,λ2ԐHbo2,λ2−1ΔOD(Δt,λ1)/DPFλ1ΔOD(Δt,λ2)/DPFλ2

In the equation, Δ*OD* represents the change in optical density, which is calculated from the known incident light intensity and the detected transmitted light intensity. Ԑ repsilonε refers to the wavelength-dependent molar absorption coefficient, which can be obtained from the relevant literature [35]. DPF stands for the differential path length factor, with ${\mathrm{DPF}}_{\lambda 1}$ set to 6 and ${\mathrm{DPF}}_{\lambda 2}$ set to 5.5 in this study. L represents the geometric distance between the light source and the detector [36]. The results are presented as time-varying curves for HbO_2_ (red curve) and HbR (blue curve), which fully characterize the hemodynamic response of the brain triggered by the Valsalva maneuver.

It should be noted that, although the system hardware supports the acquisition of short-separation channels, the current data processing pipeline focuses primarily on the basic conversion and task-related analysis of long-channel signals. Short-separation signals have not yet been incorporated into regression models to remove superficial physiological interference. This will be a key focus of subsequent algorithmic optimization.

### 3.2. Arterial Occlusion Experiment

To evaluate the system’s capability to detect changes in HbO_2_ and HbR, two basic experimental paradigms were conducted. Three right-handed healthy male subjects (mean age: 26 ± 1 years) were recruited for a forearm reactive hyperemia experiment. A sphygmomanometer cuff was applied to the proximal elbow of the subject’s dominant arm and inflated to 220 mmHg (exceeding individual systolic blood pressure). The pressure was maintained for 30 s to completely block arterial blood flow, then rapidly released to induce local tissue ischemia–reperfusion response [17]. Subsequently, a standardized Valsalva maneuver was performed, during which subjects held their breath and exhaled forcefully for 30 s to increase intrathoracic pressure, thereby inducing cardiovascular fluctuations [37]. Both experimental paradigms followed the same protocol: a 40 s baseline period (normal breathing), a 30 s task period (cuff inflation or Valsalva maneuver), two repetitions, and a 60 s recovery period.

A dual-module array was used for probe placement. The probes were fixed on the surface of the forearm radial muscle group and the left dorsolateral prefrontal cortex (DLPFC). The LED–photodiode pair within the same module enabled short-separation measurements of 8–10 mm, primarily used to detect superficial tissue signals. Interconnection between adjacent modules formed long-separation measurement channels of 20–35 mm, resulting in a total of six long channels and four short channels. According to Monte Carlo simulations and the literature reports, a source–detector distance of 20–35 mm ensures that the detection depth reaches the cortex (approximately 10–20 mm) [33]. Throughout the experiment, subjects were required to remain at rest. Data were acquired at a sampling rate of 10 Hz, and one long channel was selected for detailed analysis.

**Figure 5:**
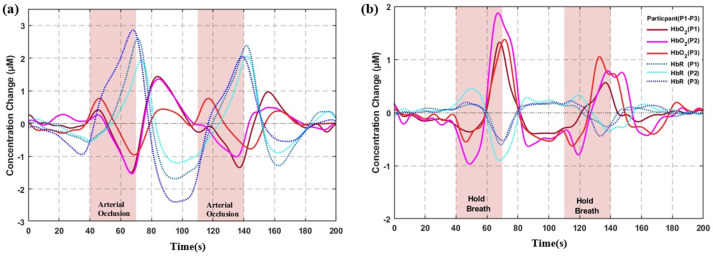
Hemodynamic responses measured by the fNIRS system: (**a**) Forearm arterial occlusion experiment; (**b**) Valsalva maneuver over the left prefrontal cortex. The red solid line represents ΔHbO_2_; the blue dashed line represents ΔHbR. The white region indicates the resting period, and the orange shaded region denotes the occlusion (220 mmHg) or breath-hold duration.

In the forearm arterial occlusion experiment, the system successfully captured local hemodynamic changes induced by blood flow blockade. The three subjects’ HbO_2_ and HbR concentration changes are shown in Figure 5a. To quantify the occlusion effect, the two occlusion periods (40–70 s and 110–140 s) were combined as the task period, and the two baseline periods (0–40 s and 70–110 s) were combined as the control period. The mean concentration during the task and baseline periods was calculated for each subject.

The results showed that, during occlusion, HbO_2_ concentration decreased by an average of 5.20 ± 5.56 μM compared to baseline, with a large effect size (Cohen’s d = 0.94), indicating a clear physiological effect. The paired *t*-test did not reach statistical significance (t(2) = 1.62, *p* = 0.247), which may be due to the small sample size (n = 3) and inter-individual variability. HbR concentration increased by an average of 10.96 ± 7.69 μM compared to baseline, with a large effect size (Cohen’s d = −1.43), and the HbR change direction was consistent across all subjects (t(2) = −2.47, *p* = 0.132). Although statistical power was limited, the observed trends were consistent with known physiological mechanisms, validating the system’s sensitivity in capturing hemodynamic changes.

### 3.3. Valsalva Experiment

Three healthy subjects (P1–P3) each performed two Valsalva maneuvers (30 s breath-holding). The system successfully captured the biphasic HbO_2_ response in the prefrontal cortex (Figure 5b). The response pattern was consistent across all subjects: HbO_2_ briefly decreased at the onset of breath-holding, then continuously increased until the end of the breath-hold, and gradually returned to baseline during recovery. Quantitative analysis of the breath-holding events showed a mean decrease in amplitude of 13.86 ± 3.85 μM (mean ± SD), a mean increase in amplitude of 38.69 ± 16.21 μM, a mean peak time of 27.10 ± 2.39 s (near the end of breath-holding), and a mean recovery time of 10.45 ± 3.05 s (returning to baseline approximately 10 s after breath-holding ended). Since all breath-holding durations were fixed at 30 s, the correlation between breath-hold duration and peak concentration could not be assessed. These parameters exhibited good consistency both within and across subjects, validating the system’s ability to reliably measure hemodynamic responses [18].

### 3.4. Working Memory Experiment

**Figure 6:**
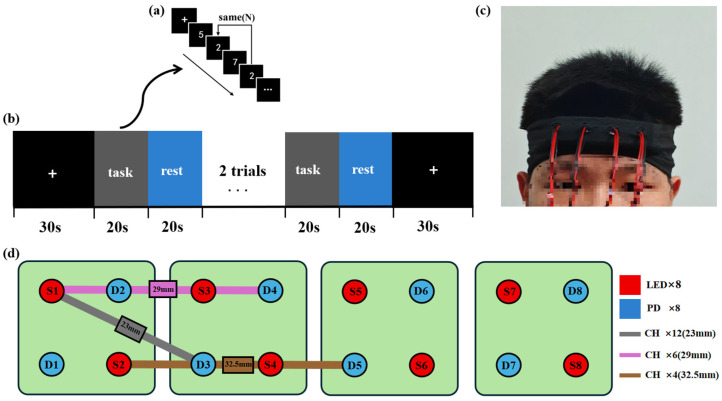
(**a**) The 2-back stimulation paradigm: participants respond when the current digit matches the one two steps prior (press “Z” if matched, “M” if not). (**b**) Experimental procedure: four continuous stimulation blocks, total duration 220 s. (**c**) Real-world setup of the working memory task. (**d**) Schematic of the light source and detector arrangement.

Working memory (WM) tasks refer to a limited-capacity system used to temporarily store information and processing resources during cognitive tasks [30,38]. The 2-back task is a commonly used working memory paradigm, recognized as an effective task to induce varying levels of cognitive load. In this study, an event-related 2-back paradigm was selected for a 26-year-old right-handed male participant [39]. In this cognitive task, the participant was presented with random numerical stimuli (ranging from 0 to 9) within a 1.5 s response window. The participant compared the current number to the previous two digits, pressing the “Z” key if they matched, and the “M” key if they differed (Figure 6a). Each load level of the task consisted of four blocks, with each block containing 12 stimuli. A 20 s rest period was given between blocks (total duration: 220 s, Figure 6b).

Based on the EEG 10-20 system, a 1 × 4 module array was placed over both hemispheres of the PFC (Figure 6c) and secured with an elastic cap, forming 22 measurement channels [40]. Among these, 12 channels had a source–detector spacing of 23 mm, 6 channels had a spacing of 29 mm, and 4 channels had a spacing of 32.5 mm (Figure 6d). The system simultaneously recorded HbO_2_ and HbR concentration changes at a sampling rate of 10 Hz to capture working memory-related cortical activation patterns.

**Figure 7:**
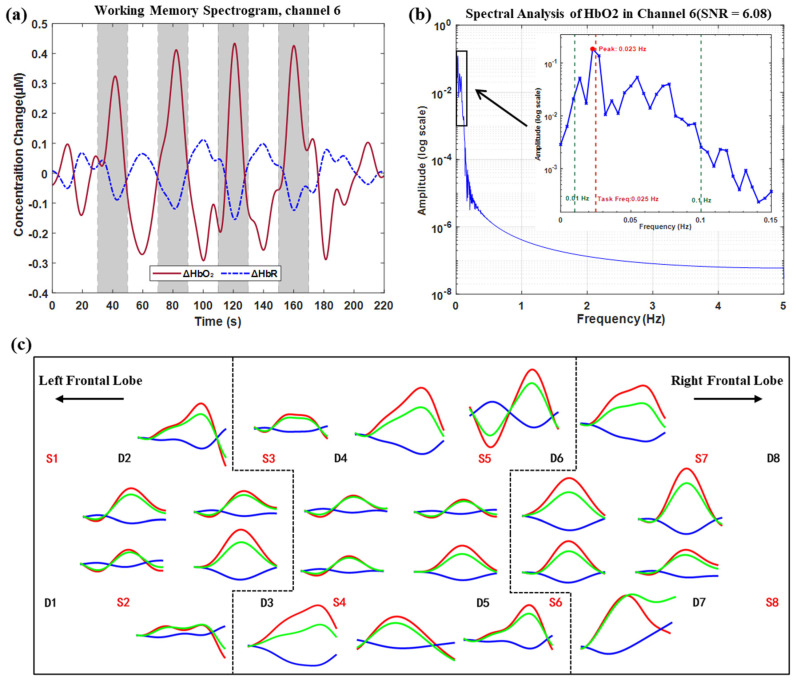
In vivo experimental results of the working memory task: (**a**) Hemoglobin concentration changes in Channel 6: red solid line (ΔHbO_2_), blue dashed line (ΔHbR). (**b**) Power spectrum of ΔHbO_2_ in Channel 6. (**c**) Block-averaged responses across all channels: red for ΔHbO_2_, blue for ΔHbR, and green for total hemoglobin (ΔHbT).

To evaluate task-evoked hemodynamic responses, the average response amplitudes across the four task blocks were calculated using the hmrR_BlockAvg function in the HOMER3 toolbox. Figure 7c shows the block-averaged responses for all 22 channels. HbO_2_ concentration began to rise approximately 5 s after task onset, peaked at 15–20 s, and then gradually declined. The mean peak time across all channels was 17.8 ± 2.3 s, consistent with the characteristics of a typical hemodynamic response function.

Based on the probe layout (Figure 7c), six pairs of left–right symmetric channels were selected (see figure caption for channel pairing). The mean activation amplitude during the task period was calculated for each channel pair. The results showed that the ΔHbO_2_ amplitude in the right PFC (0.316 ± 0.060 μM) was significantly higher than that in the left PFC (0.219 ± 0.041 μM) (paired *t*-test: t(5) = 3.23, *p* = 0.023), indicating stronger right PFC activation under high working memory load [41].

Figure 7a presents the dynamic HbO_2_ and HbR concentration responses from a representative channel (Channel 6) during the task. Figure 7b shows the power spectrum of the HbO_2_ signal from Channel 6 obtained by Fourier transform. A prominent peak was observed at 0.0227 Hz, close to the task stimulation frequency (0.025 Hz), with a signal-to-noise ratio (SNR) of 6.08. To assess the spatial distribution of this frequency component, power spectrum analysis was performed on the block-averaged HbO_2_ responses of all 22 channels. The number of channels exhibiting significant peaks within the task-related frequency band (0.02–0.03 Hz) was counted. Results showed that 72.7% (16/22) of channels displayed significant peaks near 0.0227 Hz, with the highest consistency in the right PFC region (6/6 channels). This indicates that the observed frequency component reflects a widespread task-related oscillation rather than an isolated phenomenon in a single channel. These results demonstrate that the proposed fNIRS system can effectively capture neurovascular coupling responses in the prefrontal cortex during working memory tasks.

## 4. Discussion

This study presents and validates a modular wearable fNIRS system. The system utilizes compact, lightweight optoelectronic components, with each module weighing 6.6 g. The small module size reduces the risk of discomfort during wearing and minimizes motion artifacts. The system supports either single-module operation or cascaded multi-module configurations depending on application requirements. When four modules are connected, the system provides eight short-separation and 22 long-separation channels, achieving a channel density of 1.875/cm^2^. This channel density is relatively high for wearable fNIRS systems and approaches the spatial resolution of diffuse optical tomography (DOT) [40]. It should be noted that the detection depth of fNIRS is primarily constrained by strong light scattering in biological tissues. Typical penetration depth is limited to 1–1.5 cm below the cortical surface, making reliable detection of deeper brain activity challenging [42]. Compared to the millimeter-scale spatial resolution of fMRI (typically 2–3 mm), the spatial resolution of the proposed system—and fNIRS technology in general—remains lower, but is sufficient to distinguish major brain lobes and functional subregions such as the left and right prefrontal cortices.

The time-division multiplexing architecture introduces an inherent trade-off between channel count and temporal resolution. As described in Section 2.2, the system frame rate decreases linearly as the number of modules N increases, resulting in a theoretical frame rate of approximately 7.14 Hz for the full 28-module configuration—the lower limit of the configurable range. For most task-based fNIRS studies, where hemodynamic response frequencies are typically below 0.1 Hz, a 7.14 Hz sampling rate is sufficient and allows effective separation of physiological noises such as heartbeat and respiration. However, for studies requiring high temporal resolution (e.g., fast event-related designs), this frame rate may become a limiting factor. In such cases, researchers can reduce the number of modules to achieve a higher sampling rate or adopt high-density coverage in specific brain regions while sacrificing whole-brain coverage.

The hardware design supports simultaneous acquisition of short-separation (8–10 mm) and long-separation (20–35 mm) channels, providing the physical foundation for removing superficial physiological interference. In the current data processing workflow, short-channel signals have not yet been incorporated into regression models. Short-channel regression techniques, such as nuisance regression based on the general linear model, have been shown to effectively suppress superficial interferences including scalp blood flow, heartbeat, and respiration, thereby improving the signal-to-noise ratio and specificity of cortical signals [43].

Regarding optode-scalp coupling, the quartz light guide design achieves 93.4% transmittance in the near-infrared band. It avoids heat accumulation through non-contact coupling and simplifies disinfection procedures. However, its rigid structure may introduce micro-motion artifacts in dynamic scenarios, and the 10 mm protrusion from the housing may cause discomfort in subjects with small head circumferences. Future work will optimize the light guide tip geometry (e.g., rounded edges) and explore adjustable-length designs. In recent years, hydrogel-based flexible interface materials have gained attention due to their optical transmittance, skin conformality, and biocompatibility [44,45]. Replacing rigid light guides with hydrogel-based optical layers could further suppress motion artifacts and enhance long-term wearing comfort.

Compared to commercial and research-grade wearable fNIRS platforms, the proposed system offers advantages in channel configuration (flexible expansion from 16 to over 64 channels), sampling rate (configurable from 7.14 to 200 Hz), and lightweight design (four modules < 200 g). Most commercial systems, such as the Artinis Brite MKIII (27 channels, 300 g) and NIRSport2 (up to 45–55 channels, 900 g), have fixed channel counts and limited reconfigurability. The modular design of this system provides a flexible solution for dynamic scenarios and diverse research applications.

Preliminary human experiments demonstrated the physiological detection capability of the system. In the forearm arterial occlusion experiment, HbO_2_ decreased by 5.20 ± 5.56 μM (Cohen’s d = 0.94) and HbR increased by 10.96 ± 7.69 μM (Cohen’s d = −1.43), with large effect sizes and consistent direction of HbR changes across subjects. In the Valsalva experiment, the system successfully captured the biphasic response, with a mean decrease amplitude of 13.86 ± 3.85 μM and a mean increase amplitude of 38.69 ± 16.21 μM. In the 2-back working memory task, right PFC activation was significantly higher than left PFC activation (0.316 ± 0.060 μM vs. 0.219 ± 0.041 μM, *p* = 0.023), and 72.7% of channels exhibited task-related oscillations at 0.0227 Hz. These results demonstrate the system’s sensitivity in capturing hemodynamic changes and its capability to resolve spatial heterogeneity in brain function.

In summary, the proposed system offers a combination of modularity, lightweight design, and reliable signal quality, providing a robust platform for wearable fNIRS applications in cognitive neuroscience, clinical rehabilitation, and naturalistic settings.

## 5. Conclusions

This paper presents a modular wearable fNIRS imaging system. The core innovation lies in its cooperative imaging architecture, which allows adjacent modules to jointly form a high-density measurement network, overcoming the channel count limitations of traditional modular systems. Additionally, each module integrates short-separation detection channels internally, while inter-module cascading creates long-separation channels for probing different cortical depths. The quartz light guide-based optical coupling structure enhances signal quality while ensuring wearing safety. Experimental results demonstrate that the system can reliably capture dynamic changes in HbO_2_ and HbR concentrations, offering a robust solution for brain function monitoring in naturalistic settings.

## Data Availability

The data presented in this study are available upon request from the corresponding author due to privacy protection for subjects.
